# Gestational diabetes mellitus prevalence in Maela refugee camp on the Thai–Myanmar Border: a clinical report

**DOI:** 10.3402/gha.v7.23887

**Published:** 2014-05-12

**Authors:** Mary Ellen Gilder, Thet Wai Zin, Nan San Wai, Ma Ner, Paw Si Say, Myint Htoo, Say Say, Win Win Htay, Julie A. Simpson, Sasithon Pukrittayakamee, Francois Nosten, Rose McGready

**Affiliations:** 1 Shoklo Malaria Research Unit, Mahidol-Oxford Tropical Medicine Research Unit, Faculty of Tropical Medicine, Mahidol University, Mae Sot, Thailand; 2 Centre for Molecular, Environmental, Genetic and Analytic Epidemiology, Melbourne School of Population and Global Health, The University of Melbourne, Melbourne, Australia; 3 Faculty of Tropical Medicine, Mahidol University, Bangkok, Thailand; 4 Nuffield Department of Medicine, Centre for Tropical Medicine, University of Oxford, Oxford, UK

**Keywords:** gestational diabetes mellitus, refugee, prevalence, Myanmar

## Abstract

**Background:**

Individuals in conflict-affected areas rarely get appropriate care for chronic or non-infectious diseases. The prevalence of gestational diabetes mellitus (GDM) is increasing worldwide, and new evidence shows conclusively that the negative effects of hyperglycemia occur even at mild glucose elevations and that these negative effects can be attenuated by treatment. Scientific literature on gestational diabetes in refugee camp settings is critically limited.

**Methods:**

A 75 g 2-hour glucose tolerance test was administered to 228 women attending the antenatal care (ANC) clinic in Maela refugee camp on the Thai–Myanmar border. Prevalence of GDM was determined using the HAPO trial cut-offs [≥92 mg/dL (fasting),≥180 (1 hour), and≥153 (2 hour)] and the WHO criteria [≥126 mg/dL (fasting), and 140 mg/dL (2 hour)].

**Results:**

From July 2011 to March 2012, the prevalence of GDM was 10.1% [95% confidence interval (CI): 6.2–14.0] when the cut-off determined by the HAPO trial was applied. Applying the older WHO criteria yielded a prevalence of 6.6% (95% CI 3.3–9.8). Age, parity, and BMI emerged as characteristics that may be significantly associated with GDM in this population. Other risk factors that are commonly used in screening guidelines were not applicable in this diabetes-naïve population.

**Discussion:**

The prevalence of GDM is lower in this population compared with other populations, but still complicates 10% of pregnancies. New evidence regarding gestational diabetes raises new dilemmas for healthcare providers in resource-poor settings. Efforts to identify and treat patients at risk for adverse outcomes need to be balanced with awareness of the risks and burdens associated with over diagnosis and unnecessary interventions. Screening approaches based on risk factors or using higher cut-off values may help minimize this burden and identify those most likely to benefit from intervention.

Gestational diabetes mellitus (GDM), defined as glucose intolerance developing or first recognized during pregnancy, has been identified worldwide as an increasingly common complication of pregnancies. This is a new threat to the health of women in low- and middle-income countries (1) and refugee populations, where obesity and overweight are emerging (2). There are very few published studies on GDM in the refugee camp setting, although the need for services to manage chronic diseases, including diabetes, in refugee settings has been recognized (3).

Recent solid evidence for the negative impact of GDM on pregnancy and the benefit of treatment comes from the Hyperglycemia and Adverse Pregnancy Outcomes (HAPO) study: a large multinational prospective study that included 25,505 women in the third trimester of pregnancy (4). The frequency of the primary outcomes (primary cesarean delivery, clinical neonatal hypoglycemia, and birth weight or cord serum C-peptide above the 90th percentile) and secondary outcomes (premature delivery, shoulder dystocia or birth injury, intensive neonatal care, hyperbilirubinemia, and preeclampsia) increased with increasing maternal glucose levels without a clear threshold effect. Notably, these associations were detected even for blood glucose levels that were previously considered normal and did not differ among the 15 centers in nine countries that participated in the study. Published a few years earlier, the Australian Carbohydrate Intolerance Study in Pregnant Women (ACHOIS) trial demonstrated convincingly that treating mild gestational hyperglycemia resulted in improved outcomes (5).

This has led to recommendations for adopting more sensitive screening tests, but controversy remains. The United States has adopted a universal screening policy, whereas the UK, Australia and Thailand, among others, screen based on risk factors (6). Screening programs in developing countries are even more ad hoc and heterogeneous (7). The risk factors that are included in screening guidelines may differ from country to country but often include family history of diabetes, history of previous macrosomic baby, obesity, high-risk ethnicity, GDM in previous pregnancy, and advancing age.

In 1999, the WHO recommended thresholds for a 2 hour 75 g glucose tolerance test of ≥126 mg/dL (fasting) and 140 mg/dL (2 hour), with a screening test considered positive if a single value is elevated (8). The HAPO study (4) and the International Association of Diabetes Pregnancy Study Group (IADPSG) (5) proposed the thresholds of ≥92 (fasting), 180 (1 hour), and 153 mg/dL (2 hours) (4, 9). Still other, less widely recognized, cut-offs have been proposed in the literature (9–11). An often-cited review by Ryan et al. (11) suggested screening cut-offs that would give a twofold increased risk of fetal macrosomia (fasting ≥95 mg/dL, 1 hour≥191 mg/dL, and 2 hour≥162 mg/dL). Until very recently, the United States continued to use a two-step screen with a 50 g and 100 g glucose challenge, further confounding efforts to make international comparisons (6, 7).

Critics have questioned the benefit of increasingly sensitive GDM screening tests that may promote interventions, with their inherent risks, even in mild cases where intervention will cause only a marginal reduction in adverse outcomes (7, 9, 11–13). In addition, strategies developed to date for GDM screening and testing have not taken into account feasibility and applicability for resource-poor settings (7).

Starting in 2011, the Shoklo Malaria Research Unit (SMRU) clinic in Maela refugee camp began a pilot diabetes screening program to try to address the increasing prevalence of overweight in our antenatal care (ANC) population. The percent of ANC patients with high-risk body mass index (BMI), defined as BMI ≥27.5 kg/m^2^, has increased from 4.2% in 2003–2005 to 7.9% in 2009–2012. This increase in overweight has been a consistent trend despite ongoing rates of underweight (BMI <18.5 kg/m^2^) around 10% in the same population.

Recently published research in the neighboring Thai Karen villages in Thasongyang, Thailand, showed a 16.72% prevalence of impaired fasting glucose (≥100 mg/dL). This was significantly higher in women compared with men (21.02 vs. 10.57, *p*<0.05) (14). The same group also demonstrated that overall knowledge about diabetes is very low in this population, and is significantly lower among women than men (15). Only 27.1% of survey respondents of either gender knew that pregnant women were at risk for diabetes.

The objective of this analysis is to estimate the proportion of pregnant women in a refugee camp affected by GDM based on the first results of this pilot screening program. Secondary objectives included analysis of baseline characteristics associated with GDM and outcomes. Our hypothesis was that the prevalence of GDM in this refugee population would be lower than in other Asian populations, but that associated characteristics and outcomes associated with GDM would be similar. This hypothesis was based on the fact that underweight and undernutrition remain significant health issues in the refugee population (16). This coexistence of obesity and undernutrition in a single population, known as the nutritional transition, has been described throughout the developing world (17).

The results will help inform guidelines for diabetes screening in this and neighboring pregnant populations.

## Methods

### Setting

About 140,000 refugees from Myanmar have been housed in refugee camps along the Thai–Myanmar border since the 1980s (18). The Shoklo Malaria Research Unit (SMRU) provides the antenatal and delivery care for the population of the Maela refugee camp, the largest of nine refugee camps in western Thailand. Approximately 90% of pregnant women in the refugee camp receive ANC at the SMRU clinic (19). The patient population consists of 45–50,000 people of multiple ethnic groups, predominantly Karen, who have been displaced from their homes in Myanmar. A limited number of jobs are available within the camp in various social services (health, education, child protection, etc.) and remittances are sent back to the camp from relatives working in Thailand or abroad. Rations of rice, dried fish, and chili are provided, with additional rations of a fortified flour mix, oil and dried peas for children under five, older children who are malnourished, and pregnant women. These rations are being gradually decreased due to funding cuts, and are dominated by carbohydrates. At the same time, the informal markets within the camp are expanding and some vegetables, meat and various prepared foods are available for purchase, sold by camp residents. Some families have small kitchen gardens at their houses, but space is limited. Camp residents are forbidden from traveling outside the camp without pre-arranged permission with camp authorities. As a result, farming and foraging for locally known wild vegetables, two activities that would traditionally provide exercise and improved nutrition for women, are limited.

Individuals living outside the camp in villages in Myanmar or Thailand who share the same languages also access SMRU's delivery room. The clinic has a delivery room that provides delivery services for about 1,500 women per year but does not have insulin or the capability to perform cesarean section on site. Patients requiring cesarean delivery are transported for about an hour to the closest emergency obstetric services in Thailand at Mae Sot Hospital. SMRU also provides a similar service for migrant women in two settings outside the refugee camp.

General medical care in the camp is provided by Première Urgence – Aide Médicale Internationale and there is currently no published data on the prevalence of diabetes in the general camp population.

From 5 July 2011 to 6 March 2012, women receiving ANC in the SMRU clinic in Maela camp were invited to come for diabetes screening at between 24 and 28 weeks of gestation. Due to staff time constraints, a maximum of two patients were recruited per day for this pilot program. The staff was instructed to invite consecutive patients based on staff availability and not on patient characteristics. Unfortunately, the total number of patients invited to come was not recorded, and we cannot quantify the response rate. Capillary blood glucose levels were measured when fasting, and then 1 and 2 hours after ingestion of 75 g of anhydrous glucose dissolved in 200 ml of water. Capillary sampling has been shown to be a cost-effective alternative to venous sampling in low-resource settings (20).

### Sample size calculation

A sample size of 216 was the minimum needed to estimate the prevalence of GDM in this population, assuming a prevalence of around 10% with a confidence interval of ±4%. Analysis was performed on the first 228 completed tests. This included an additional 12 patients (~5%) to account for the possibility of drop-out from ANC before pregnancy outcome was known.

### Data extraction

All birth records from July 5, 2011, to March 6, 2012, were audited and the data were extracted and anonymized for analysis.

### Ethical approval

Ethical approval was given by the Oxford Tropical Research Ethics Committee for the use of the antenatal records (OXTREC 28-09).

### Statistical analysis

Data were analyzed using Stata version 12.0 (StataCorp, College Station, TX, USA). Continuous normally distributed data were described using the mean and standard deviation, and non-normally distributed data using the median and range. Categorical data were summarized using frequency and percent. Means were compared with the Student's *t*-test, medians with Mann–Whitney U test and proportions with the Chi-squared test. Logistic regression was used to evaluate the interactions between variables, but this was limited by the small sample size.

## Results

The first 228 patients with completed screening test results available were included in the analysis.

### Prevalence of GDM

Analysis of the 228 oral glucose tolerance test results using the HAPO criteria (4) yielded a GDM prevalence of 10.1% (23/228) (95% CI 6.1–14.0); 6.6% (15/228) (95% CI 3.3–9.8) using the older WHO criteria (7) and 5.3% (12/228) (95% CI 2.3–8.2) with criteria proposed by Ryan et al. (11). Ryan's criteria identified about half of those who would be identified using the stricter HAPO criteria (12 of the 23). The WHO criteria, on the contrary, concurred less well with the HAPO results. WHO criteria identified five individuals as having GDM who were not identified using HAPO criteria and failed to identify 13 women who were identified by HAPO.

### Patient characteristics associated with GDM

Information on baseline characteristics was available for all 228 women tested. There was a significantly greater mean age and higher proportion of illiteracy in the GDM group (Table 1) although illiteracy was not statistically significant after controlling for age (*p*=0.114). Higher parity approached significance (*p*=0.05).

**Table 1:** Characteristics of women according to screening results following the 75 g oral glucose tolerance test

Risk factor characteristic	Positive*, *N*=23	Negative, *N*=205	*p*
Age, years	29 (6.3) [16–43]	26 (6.5) [15–46]	0.039
Parity (median)	2 [0–5]	1 [0–9]	0.050
BMI at 22 weeks gestation, mg/kg^2^ (median)	24.2 [20–32.4]	23.2 [16–39.6]	0.454
Proportion overweight (BMI≥27.5 kg/m^2^)	4/23 (17.4%)	16/205 (7.8%)	0.123
Literacy, *n* (%)	12/23 (52.2)	151/205 (73.7)	0.030
Previous cesarean section, *n* (%)	2/23 (8.7)	8/204 (3.9)	0.290

Data are reported as mean (SD) [range] unless otherwise stated.

* HAPO criteria used.

Other variables such as ethnicity (multiple ethnic groups from Myanmar are represented in the camp), blood pressure, fundal height, history of stillbirth, and country (Thailand or Myanmar) of birth were found not to be associated with GDM in this cohort. Likewise historical criteria (e.g. family history of diabetes, personal history of pre-diabetes) used in some countries to select pregnant women for screening due to increased risk associated with these factors, were not associated with GDM in this setting. There was no statistically significant increase in BMI or history of previous cesarean section. The small number of GDM in this study precluded a formal risk factor analysis.

### Birth outcome

In the cohort of 228 pregnant women, delivery outcomes were known for 221 patients, including all patients who screened positive for GDM. Seven women were lost to follow-up before delivery and those without known outcomes were excluded from the subsequent analysis. These 221 outcomes included normal vaginal delivery 90.5% (200/221): at SMRU (180) or at a Thai Hospital (7) or at home (13); vaginal breech delivery at SMRU 1.8% (4), vacuum delivery at SMRU 1.4% (3), and cesarean section delivery at the Thai Hospital 6.3% (14).

Of the 212 newborns with birth weight measured in the first 24 hours, the mean birth weight for the GDM group was 3,175 g (range 2,050–3,900) compared with 3,036 g (range 1,500–4,230) in the screen normal group (*p*=0.11). Of the five infants born weighing 4 kg or more in this cohort, none were born to women who screened positive for GDM. There were no neonatal deaths. Two intrapartum fetal deaths occurred in non-diabetic pregnancies, one following placental abruption and the second with a complicated delivery of a second twin.

The proportion of cesarean delivery (as the outcome for the current pregnancy) was 13.0% (3/23) in diabetic patients and 6.1% (12/198) in non-diabetic patients, (*p*=0.360). There was no significant difference in estimated gestational age at delivery (39.3 weeks for GDM group *vs*. 39.0 weeks non-diabetic group, *p*=0.24) or APGAR scores at 1 and 5 min (median 9, 10 for both groups). Six infants, all in the non-diabetic group, required resuscitation and resuscitation data were not available for one diabetic woman, who underwent cesarean delivery at the Thai hospital.

## Conclusions

This is the first report of GDM prevalence in a refugee camp using HAPO criteria. Refugee healthcare is often based on emergency responses and chronic, non-infectious diseases such as diabetes are often poorly addressed due to lack of perceived urgency as well as cost and very real logistical difficulties in providing long-term continuity of care. The low prevalence of 10% is not surprising in a refugee population where nutrition remains an issue (16) and micronutrient deficiency of thiamine was the most common cause of infant deaths just two decades ago (21). However, a recent review of GDM prevalence worldwide showed Southeast Asian populations to have the highest prevalence overall, with an average 23% (HAPO criteria) of pregnancies affected (22). Management of gestational diabetes involved exogenous insulin in only 8–20% of women in two recent large multi-center treatment studies [the Maternal-Fetal Medicine Units (MFMU) study (23) (23) and the ACHOIS study (5)] so the great majority of women with gestational diabetes have improved outcomes with diet, lifestyle modification, oral hypoglycemic drugs and glucose monitoring alone. A study of GDM in the United Nations Relief and Works Agency (UNRWA) clinics of Gaza showed significant improvement of outcomes with an educational intervention (24). These noninvasive treatments are potentially available in low-resource settings and could reduce the risk of cephalo-pelvic disproportion and shoulder dystocia, and need for cesarean section.

On the other hand, implementing a universal screening strategy in this setting comes with some inherent costs. The staff time required to administer a 2 hour test to 1,500 women per year (the average case load in the Maela ANC clinic) is significant, and like most resource-poor settings, will further burden a limited staff. In addition, it has been suggested that the HAPO criteria may yield more false-positive tests in a real world setting than they did in the carefully regimented study setting due to surreptitious or accidental non-fasting status (13). This might be especially true in our setting where literacy and health literacy are quite low. This low literacy means that effective counseling on diet and exercise will be more time intensive, as patient handouts are of limited use. Increasing the rate of cesarean deliveries by inappropriately assigning high-risk status to low-risk patients can lead to serious risks in subsequent pregnancies, especially in a mobile population that frequently lack access to appropriate emergency or operative obstetric services.

Taking all of these factors into account, we tentatively suggest that a risk-factor-based screening might identify the patients at highest risk for adverse outcomes without unnecessarily burdening staff and patients with unnecessary testing.

This analysis is limited by several factors. Since this screening was conducted in a clinical setting without supervision of the women the night before the test, fasting status of the patients involved could not be guaranteed. As the exact percentage of patients who were invited for screening was not recorded, we cannot exclude the possibility of selection bias. However, the very low level of local awareness of GDM (15) makes staff selection or self-selection of high-risk patients unlikely. Finally, the small sample size limits the strength of outcome analysis and precludes formal risk factor analysis.

Despite these limitations, analysis of this cohort sheds some light on characteristics that could be used to identify patients in this population at higher risk for an abnormal screening test result, potentially conserving resources and reducing the number of false-positive results. As expected, it appears that high parity and maternal age are associated with GDM in this population. Though we did not show a statistically higher BMI in the GDM group, long-term outcomes of infants of mothers with GDM and obesity are worse than infants of mothers with GDM and normal BMI (8). For this reason, it would be logical to include obese women in screening algorithms.

The lack of association with features of history, family history of GDM and personal history of glucose intolerance, is expected in a setting where chronic under diagnosis and under treatment of diseases like diabetes are combined with low health literacy. This was likewise reported in a recent review on the practicalities of GDM screening in resource-poor settings (7). However, these historical risk factors may become more useful in the future if screening and educational programs increase local awareness of diabetes. In the UNRWA clinics in Gaza Strip, where non-communicable diseases are the primary health focus, prior macrosomia or family history of diabetes was strongly correlated with GDM (25). In addition, prior history of abortion or of stillbirth was a significant risk factor in their population. In our population, these were no different in the two groups, possibly because of a higher rate of infectious causes of stillbirth and abortion. Prior history of cesarean delivery was also a significant risk factor in the UNRWA clinics. In our population, the women with GDM were twice as likely to have a previous history of cesarean delivery, but in our small sample this was not significant (Table 1). A history of previous cesarean section may be a surrogate marker for previous undiagnosed GDM, and these patients should be screened.

Diabetes has generally been considered a disease of abundance, but this report shows it complicates one in 10–20 pregnancies in this refugee situation, depending on the diagnostic criteria applied. In 2010, the UNHCR (26) estimated that 15.4 million individuals have been forced to cross international borders to become refugees, and to our knowledge GDM screening has been described in only one other refugee camp setting (Gaza Strip) (24, 25). Despite the weight of evidence for the benefits of early diagnosis and treatment of GDM, the absence of a simple, inexpensive and applicable screening method remains a major barrier to GDM screening programs in refugee camps and other resource-poor settings.
